# Long sequence temporal knowledge tracing for student performance prediction via integrating LSTM and informer

**DOI:** 10.1371/journal.pone.0330433

**Published:** 2025-09-09

**Authors:** Ailian Gao, Zenglei Liu

**Affiliations:** School of Electrical and Information Engineering, Hunan Institute of Technology, Hengyang, Hunan, China; University of Education, PAKISTAN

## Abstract

Knowledge tracing can reveal students’ level of knowledge in relation to their learning performance. Recently, plenty of machine learning algorithms have been proposed to exploit to implement knowledge tracing and have achieved promising outcomes. However, most of the previous approaches were unable to cope with long sequence time-series prediction, which is more valuable than short sequence prediction that is extensively utilized in current knowledge-tracing studies. In this study, we propose a long-sequence time-series forecasting pipeline for knowledge tracing that leverages both time stamp and exercise sequences. Firstly, we introduce a bidirectional LSTM model to tackle the embeddings of exercise-answering records. Secondly, we incorporate both the students’ exercising recordings and the time stamps into a vector for each record. Next, a sequence of vectors is taken as input for the proposed Informer model, which utilizes the probability-sparse self-attention mechanism. Note that the probability sparse self-attention module can address the quadratic computational complexity issue of the canonical encoder-decoder architecture. Finally, we integrate temporal information and individual knowledge states to implement the answers to a sequence of target exercises. To evaluate the performance of the proposed LSTKT model, we conducted comparison experiments with state-of-the-art knowledge tracing algorithms on a publicly available dataset. This model demonstrates quantitative improvements over existing models. In the Assistments2009 dataset, it achieved an accuracy of 78.49% and an AUC of 78.81%. For the Assistments2017 dataset, it reached an accuracy of 74.22% and an AUC of 72.82%. In the EdNet dataset, it attained an accuracy of 68.17% and an AUC of 70.78%.

## Introduction

For the last few years, a large number of students have had to rapidly switch to online learning, which has caused significant changes in the learning environment and learning methods [1]. In a typical online learning platform such as Massive Open Online Courses (MOOCs) [2], it is difficult for teachers to monitor each student’s learning progress in real time, like in traditional classrooms. Knowledge tracing (KT) models [3,4], as one fundamental component of an online learning platform, can provide personalized learning resources and paths based on each student’s learning activity and performance while improving learning efficiency. Teachers can use the KT algorithms to track students’ learning progress and understanding level in real time, enabling them to timely master the students’ knowledge status. By analyzing students’ historical learning data, KT can generate predictions of students’ future learning performance, thereby helping teachers and educational institutions develop intervention measures in advance and prevent students from falling behind in learning. However, it is still difficult to directly evaluate the capabilities of one specific KT method. Instead, the proficiency level of a student’s knowledge is typically assessed by the KT models through forecasting the student’s next exercise outcome based on their historical responses to exercises.

Most existing KT models focus on short-sequence prediction but fail to model long-term learning trajectories, which are critical for understanding sustained knowledge mastery and designing timely interventions. In addition, these models often overlook temporal dynamics and struggle with computational inefficiency when handling long sequences. Bearing the above-mentioned analysis in mind, this study propose a novel KT pipeline, dubbed the Long Sequence Temporal Knowledge Tracing Method for student performance prediction (LSTKT). The goal of this model is to accurately track the learner’s level of knowledge by combining the Bidirectional Long Short Term Memory (Bi-LSTM) [25] with Informer model [26]. The proposed method uses a Bi-LSTM network to record how students’ learning activities change over short periods of time. The Informer network, on the other hand, creates long-term sequence data and pulls out deep temporal features. To be specific, the input layer of our model can receive data from students interacting with the online platforms, including question answer sequences and timestamps. We then exploit the Long Short Term Memory (LSTM) networks to process input sequences and capture short-term dynamic changes in the students’ knowledge states.

Additionally, the Informer model receives the LSTM layer’s output as input, utilizing the self-attention mechanism to handle long sequence data and extract long-term dependencies. Using feature fusion, we combine the short-term features captured by the LSTM layer with the long-term features of the Informer layer to form a comprehensive feature representation. One or more fully connected layers map the detailed features to the output space in the final part of the proposed model. This lets us guess how likely it is that a long string of students’ answers to the exercises will be correct. The Informer model employs a probabilistic sparse self-attention mechanism. This can greatly simplify computations while still maintaining long-term temporal information. The cross-entropy loss function and Adam optimizer train the entire model, while the back-propagation algorithm updates the weighting parameters.

In general, this work’s contributions can be summarized as follows:

- This study proposes a novel KT model that comprehensively leverages long-sequence time-series information captured from students’ exercising records.
- To implement the proposed algorithm, both the bidirectional LSTM and Informer models are leveraged.
- Extensive experimental results on the publicly available datasets demonstrate the superiority of the proposed approach over state-of-the-art methods.

We have structured the remaining sections of the paper as follows: Sect describes the related works to this study. Sect provides a comprehensive explanation of the suggested methodology aimed at resolving the long-sequence time-series knowledge tracing task. Sect provides the experimental results of the comparison experiments. It provides details about the leveraged datasets, the evaluation metrics used, and the hyper-parameter settings. Sect gives the discussion about the outcomes of the proposed approach in this work. Finally, the conclusion is provided in Sect, which discusses the future research direction.

## Related work

### Traditional knowledge tracing methods

The literature has proposed numerous KT methods over the past few decades. Early KT algorithms are based on simple machine learning models, such as the random forest [5], the Bayesian network [6], and the Markov model [7]. For instance, as an early work in the field of KT, Bayesian KT (BKT) [8] proposed an effort to model students’ varied knowledge states during knowledge acquisition. This study denotes unobserved states of students’ knowledge acquisition as hidden variables in a Hidden Markov Model (HMM), which can update the posterior probabilities of students’ knowledge states using a Bayesian network based on the responses to exercises. Accordingly, the parameters of Bayesian and HMM models, like the probability of mastering a Knowledge Concept (KC) [9], can be calculated. Meanwhile, it also incorporates the probabilities of changing knowledge states, such as from not mastered to mastered. The experimental results on three benchmarks demonstrate that their constructed model can represent knowledge concepts better than state-of-the-art semantic networks. In the work of [10], a higher order Item Response Theory (IRT) model approximates students’ initial knowledge states as their one-dimensional overall proficiency while integrating the estimated difficulty and classification of each skill to estimate the probability of mastering a skill before practicing it. And then the skill-wise knowledge is exploited for tracing probabilities of learning, guessing, and slipping. The overall accuracy of this model on real data from algebra tutor is 87.13%.

### Deep learning-based knowledge tracing methods

Recently, plenty of researchers have leveraged the deep learning models to address KT tasks. For instance, Piech et al. [11] have pioneered this direction of study and rendered the capability of deep learning models for KT. In this study, the utility of Recurrent Neural Networks (RNNs) [12] was explored to model students’ learning activities. The RNN-based models had achieved superior outcomes over previous techniques without domain knowledge. It achieved area under ROC curve (AUC) of 0.82, 0.85, and 0.86 on three datasets simulated-5, Khan math, and ASSISTments, respectively. And it can also capture complicated representations of students’ knowledge. The adoption of Neural Networks (NNs) [13] results in substantial enhancements in prediction performance on a wide range of KT datasets. To make use of the associations between knowledge points in KT, Duan et al. [14] used Generative Adversarial Networks (GANs) to yield knowledge relationship representations by integrating multiple knowledge associations. In their proposed models, they used gated recurrent units to generate the students’ knowledge states. Meanwhile, they exploited an attention-based technique to learn the coefficients of various knowledge associations. The outcomes on the ASSISTments (0.776, 0.701, 0.746), Junyi (0.882, 0.829, 0.844), EdNEt (0.891, 0.809, 0.824), and KDDCup (0.726, 0.605, 0.791) datasets have achieved superior performance over the state-of-the-arts in terms of AUC, F1 score, and accuracy, respectively. To address the shortcomings of the Deep-IRT model, which combines IRT with a deep learning model for KT, Tsutsumi et al. [15] proposed an updated Deep-IRT that can model students’ responses to an item using two different networks, including the student network and the item network. This work also presented a novel hyper-network architecture that balances both current and past data samples of students’ knowledge states. Results on six datasets demonstrate that this model can improve the prediction accuracy by about 2.0%. Furthermore, graph-based deep learning models, such as [16,17], are proposed for KT. In the work of [16], the authors proposed a heterogeneous graph-based KT method by introducing spatial-temporal evolution, in which knowledge state changes can be tracked along with both spatial and temporal dimensions. The authors leveraged the hierarchical structure to provide ample exercise representations within the knowledge space. We integrated the content, concepts, and challenges into the presented heterogeneous graph. The proposed graph network exploited both a spatial and a temporal updating module. Experiments on three datasets show the superior performance of this model in terms of performance prediction. For instance, by using the heterogeneous graph embedding, the AUC and accuracy of graph knowledge tracing model [18] on ASSISTments2015 dataset can be enhanced from 0.6901 and 0.7269 to 0.6973 and 0.7263. Sun et al. [19] proposed a KT method called weighted heterogeneous graph-based three-view contrastive learning framework, inspired by data-driven paradigms. Generally, the researchers exploited three different encoders to complement each other and obtain exercise embeddings. In particular, it thinks that the semantic information of more complex, different, and later tasks on a mixed graph can be used to make useful models. Finally, they adopted a meta-path-based positive sample choice strategy and joint contrastive loss to yield optimal prediction performance. It has achieved AUC and accuracy of 0.7927 and 0.7429 on the dataset Assistments2009, AUC and accuracy of 0.7728 and 0.7043 on the dataset Assistments2017, AUC and accuracy of 0.7663 and 0.7388 on the dataset EdNet, and AUC and accuracy of 0.8933 and 0.8545 on the dataset Statics2011. The study of [72] proposes a Structure-aware Inductive Knowledge Tracing model (SINKT) that introduces Large Language Models (LLMs) to construct a heterogeneous graph of concepts and questions, which can predict student responses by integrating student knowledge states and question representations. It has achieved state-of-the-art performance on four datasets and addressing data sparsity and cold start issues in inductive KT tasks. The work of [73] presents an Alternate Autoregressive KT framework (AAKT) that treats KT as a generative process. It represents knowledge states via AR encodings on question–response sequences, which incorporates educational and exercise details as inputs. This study has outperformed baselines on four datasets in prediction metrics.

Recently, deep learning-based architectures have obtained promising performance in KT. However, there are still several challenges. First of all, most of the existing studies focus on short-sequence time-series student performance prediction, such as predicting the student’s answer to the next question [3]. On the contrary, long-sequence time-series forecasting is more valuable than short-term time-series prediction [20]. On one hand, analyzing student performance over an extended period allows KT models to make more accurate predictions about students’ performance. Note that each student’s performance prediction is also a long sequence, such as predicting a group of answers for the next few questions. On the other hand, it can provide a holistic view of a student’s learning trajectory, revealing long-term trends and patterns for students to master knowledge [21]. Meanwhile, long sequence data samples help KT algorithms identify individualized learning paths and offer tailored resources and recommendations. Moreover, long-term data can inform the design of more effective long-term learning interventions. Intuitively, teachers should not make hasty evaluations of students’ knowledge mastery based on the students’ short-term performance [22]. Instead, most of the teachers would pay more attention to their performance over a long period of time [23]. Based on the long sequence of time-series exercise-answering records, the teacher can comprehensively take the students’ performance tendencies into consideration. Using longitudinal tracking [24] to assess the stability of a student’s knowledge state can determine whether concepts are truly mastered. In addition, the deep learning-based algorithms have been extensively employed in various prediction-related tasks, including renewable energy [63], material performance [64], biomedicine [65–69].

To be specific, we can divide the current deep learning-based KT methods into five categories, including memory structures, attention mechanisms, graph representation learning, textual features, and forgetting features.

#### Memory structures.

This type of deep learning model was inspired by memory-augmented neural networks [27]. These KT models have been enhanced by increasing more powerful memory structures, like key-value memory [28], for dynamically extracting knowledge states at a fine-grained level, including the mastery extent of every single skill. For instance, the work of [29] proposes a deep learning model-based KT model, namely Sequential Key-Value Memory Networks (SKVMN). This model exploits recurrent modeling and memory capacity for examining student learning behaviors. Sun et al. [30] propose an exercising record representation algorithm that integrates the features of learning activities along with the learning abilities, thereby enhancing the performance of KT.

#### Attention mechanisms.

Inspired by the transformer model [31] and its relative applications in the natural language processing area, a variety of attention mechanisms have been presented in deep learning KT models for capturing the relationships among exercises and the students’ knowledge status. For instance, Ghosh, Heffernan, and Lan [32] propose the Attentive Knowledge Tracing (AKT) model, which uses flexible attention-based neural network models with a series of components inspired by cognitive and psychometric models. AKT employs a monotonic attention mechanism related to the students’ future responses and previous answers, as well as the similarity between the responses. Pandey and Srivastava [33] propose a Relation-aware self-attention model for KT (RKT). RKT uses a self-attention layer to incorporate contextual information, which integrates both the exercise associations of textual content as well as students’ performance and the forgetting behavior by using an exponentially decaying kernel function. Choi et al. [34] propose a transformer-based model for KT, which is named after Separated Self-Attention Neural Knowledge Tracing (SAINT). SAINT is organized as an encoder-decoder structure, which deals with the exercise and response sequences, respectively. Jiang et al. [35] propose an adaptive heterogeneous graph embedding module to fully make use of the latent information within a graph. In the meantime, they designed two encoders to capture students’ engagement and knowledge, respectively. Shin et al. [36] propose SAINT+, an updated version of the SAINT model. SAINT+ is also a transformer-based KT model that addresses exercises and students’ responses separately. Meanwhile, SAINT+ has an encoder-decoder architecture, in which the encoder deals with a set of exercise embeddings and the decoder copes with the corresponding response embeddings. Moreover, SAINT+ uses two temporal embeddings to represent the elapsed time (the time taken to answer a question) and the lag time (the time period between neighboring learning activities). The work of [71] proposes a Fine-Grained KT model (FGKT) to capture actual differences among exercises and in prior knowledge, which can obtain exercise representations via knowledge concepts and designing an attention mechanism for prior knowledge relevance.

#### Graph representation learning.

Inspired by the representation capability of graph models like Graph Neural Networks (GNNs) [37], graph-based KT models have been proposed to leverage the rich structural information from graphs to flexibly model associations between questions and skills. Tong et al. [38] proposes a KT framework dubbed Structure-based Knowledge Tracing (SKT), which uses the multiple associations in knowledge to capture the influence propagation among KCs. The SKT framework considers not only the temporal influence on exercise sequence but also the spatial impact on knowledge. Hiromi, Yusuke, and Yutaka [39] propose a GNN-based KT method. It casts the knowledge state as a graph, which reformulates the KT task into a time-series node-level classification problem in the GNN model. The work of [70] proposes Knowledge Structure-aware Graph-Attention Networks (KSGAN) to utilize improved Graph Attention Networks for effective exercise representation by leveraging knowledge structure. It incorporates representation optimization into the loss function to address data sparsity.

#### Textual features.

Since the exercises might contain rich information about the skills required by the corresponding questions and the associations between questions, A set of deep learning-based KT models has exploited textual features from questions to track the knowledge states of the students. For instance, Liu et al. [40] propose a holistic approach to students’ performance prediction. To implement performance prediction, an Exercise-Enhanced Recurrent Neural Network (EERNN) is leveraged by exploring the student’s exercise recordings and the content of the exercises. In the EERNN model, each student’s state is turned into a vector and tracked with an RNN, while a bidirectional LSTM is used to extract the embedding of each exercise. In addition, the EERNNM model exploits both the Markov model and the attention mechanism. Su et al. [41] also propose an EERNN framework for student performance prediction by using students’ exercise records and the exercises themselves. A bidirectional LSTM is used to learn each exercise representation while tracking students’ knowledge states. Yin et al. [42] propose a pre-training model called QuesNet for generating question representations. QuesNet uses a unified framework to gather questions and the heterogeneous inputs into a vector. It consists of a hierarchical architecture to better describe the questions in an unsupervised manner.

#### Forgetting features.

Inspired by the learning curve theory, several recent deep learning KT models incorporate forgetting features to model students’ forgetting behaviors for KT. For instance, Chen et al. [43] propose an explanatory probabilistic model to implement the KT proficiency of students over time by leveraging educational priors. In this model, each student is denoted as a time-series knowledge vector in a unified knowledge space. Given the student knowledge vector, the learning curve and forgetting curve are taken as priors to capture the change in students’ temporal proficiency. To be specific, a probabilistic matrix factorization framework is leveraged for combining student and exercise priors. Abdelrahman and Wang [44] propose a KT model called the Deep Graph Memory Network (DGMN). DGMN incorporates the forget-gating mechanism into the attention module for capturing forgetting behaviors during the KT process. In addition, this model has the ability to extract associations between KCs from a latent graph with the students’ evolving knowledge states.

Notably, most existing approaches are predominantly designed for short-sequence prediction, while the critical need for long-sequence time-series forecasting in education remains underexplored. Long-sequence modeling is essential for understanding sustained learning trends, designing personalized interventions, and evaluating long-term knowledge mastery. Bearing this issue in mind, this study addresses these gaps through a novel framework that: 1) fuses short-term and long-term temporal modeling to capture both immediate knowledge dynamics and extended learning patterns; 2) integrates timestamp information with exercise sequences to enhance the model’s sensitivity to the temporal context of learning events; and 3) employs efficient long-sequence forecasting techniques to overcome the computational limitations of traditional attention mechanisms. By prioritizing long-sequence prediction, this study fills a key void in existing KT research. It also offers a more comprehensive and practically relevant solution for tracking student performance over extended periods.

## Materials and methods

### Problem definition

Assume the presence of a collection of exercises on an internet-based educational platform. The pupils participating on this platform are required to respond to several exercises. The KT model is utilized to monitor the students’ changing knowledge states by employing the exercise-answering sequence of students. Due to the intricate nature of learning as a cognitive process, it is challenging to accurately represent students’ true knowledge states over an extended duration. Alternatively, the KT models typically use students’ learning records to anticipate their responses to future exercises for the purpose of implementing KT evaluation.

Let’s consider a sequence of exercises and answers by a student U, denoted as $I_{u}=\{(e_{1},t_{1},a_{1},C_{1}),...,(e_{i},t_{i},a_{i},C_{i}),...,(e_{n},t_{n},a_{n},C_{n})\}$. Here, $I_{u}^{i}=(e_{i},t_{i},a_{i},C_{i})$ represents the exercise-answering record at step *i* with the time stamp *t*_*i*_. It should be noted that *e*_*i*_ represents the exercise that the student replies, *a*_*i*_ indicates the label of the related response, with 1 indicating a correct response and 0 indicating an incorrect response. *n* represents the total number of steps. Here, *C*_*i*_ represents a collection of KCs that correspond to *e*_*i*_, and it is possible for one exercise to be related to numerous KCs. $I_{n\;+\;1}^{u}$ represents a student named U at the next step, which is step *n* + 1. The exercise-answering records of $I_{n\;+\;1}^{u}$ consist of a sequence of learning activities that occurred before step *n* + 1. The exercise-answering records can be denoted as $I_{n}^{u}=\{I_{1}^{u},I_{2}^{u},...,I_{n}^{u}\}$.

### Overall structure of the proposed pipeline

**Fig 1:**
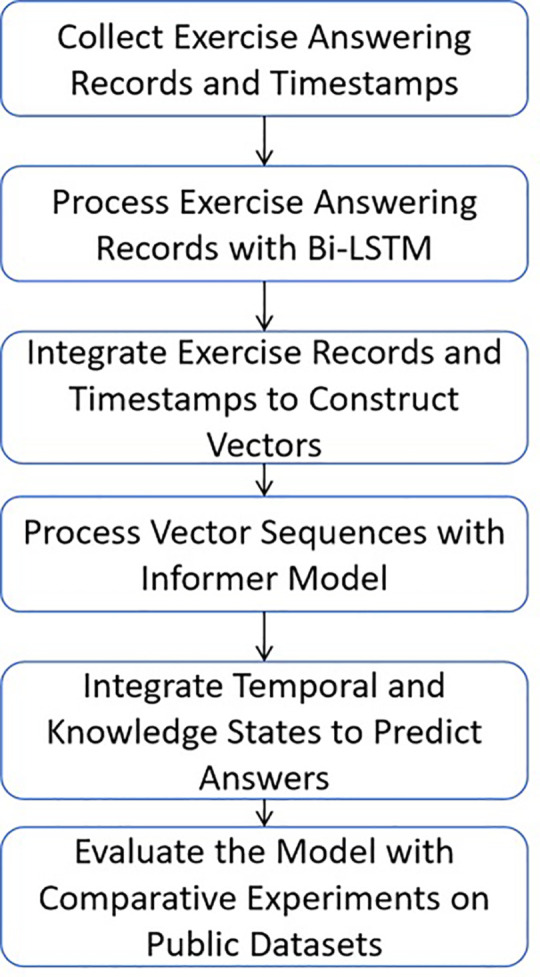
The workflow of the proposed approach.

This study proposes an innovative long-sequence time-series forecasting pipeline for knowledge tracing. As shown in Fig 1 the specific steps are as follows:

- Exercise Answering Record Embedding: The Bi-LSTM model is employed to process students’ exercise answering records. The Bi-LSTM can effectively capture the context information in the sequence and transform the exercise answering records into meaningful feature representations, providing a basis for subsequent analysis.
- Feature Vector Construction: Integrate students’ exercise records and the corresponding timestamps into a vector for each record. This integration method combines time information with exercise information, enabling the model to consider both the temporal order and specific content of students’ learning behaviors, and reflecting students’ learning states more comprehensively.
- Prediction Using the Informer Model: The constructed vector sequence is used as the input of the Informer model. The Informer model adopts a probability-sparse self-attention mechanism, effectively solving the quadratic computational complexity problem in the traditional encoder-decoder architecture. This allows the model to calculate efficiently while accurately capturing long-distance dependencies when processing long-sequence data, enhancing the prediction performance.
- Integrating Information for Prediction: By integrating temporal information and students’ individual knowledge states, predictions for a series of target exercise answers are finally achieved. This approach that takes multiple factors into account makes the prediction results better reflect students’ real knowledge mastery.

**Fig 2:**
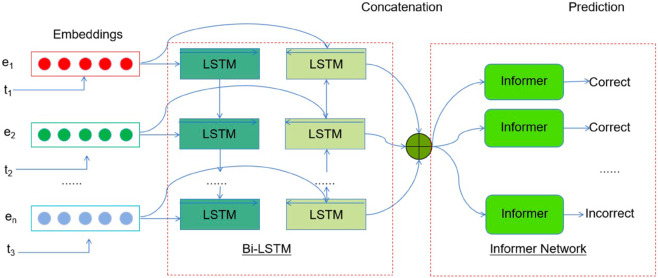
The overall framework of the proposed approach. Embedding layers: Initially, each word in the text is converted into a numerical vector using a pre-trained word embedding model, such as GloVe [45]. Input to LSTM: The numerical vectors from the word embeddings serve as the input to the LSTM model. Each vector represents a single time step in the sequence, with the sequence length being equal to the number of words in the input text.

In the following of this section, we firstly present a detailed explanation of the deep learning architecture that is being proposed. This includes a description of the learning task and the probability attention mechanism that has been suggested for knowledge tracing. During the initial stage, we employ a Bi-LSTM model to generate a robust knowledge state representation by promoting the connections between the knowledge states of students as they evolve both forward and backwards. Next, the informer network is utilized to obtain the output of the Bi-LSTM and generate the prediction results. The proposed informer model incorporates a probabilistic attention mechanism as a substitute for the conventional self-attention module. Next, an attention distillation operation is utilized to enhance dominant attention scores in stacked layers and significantly reduce the overall spatial complexity. Presented is a generative decoder that may obtain long sequence answers for exercise prediction using only one forward step, thereby eliminating cumulative mistakes in the inference process. The structure of the suggested approach is depicted in Fig 2.

### Bi-LSTM model as the embedding layers

The proposed approach employed the Bi-LSTM model as the fundamental framework. Since the introduction of the first LSTM model by Hochreiter and Schmidhuber [46], this type of deep learning algorithm has been widely used in various natural language processing (NLP) tasks and has shown promising outcomes. Multiple research in this field have shown that models based on LSTM are efficient in handling tasks associated with textual material. In addition to the exercise embeddings, the time stamps of the exercise-answering records are also included as input for the Bi-LSTM network.

This study presents a Bi-LSTM model for generating embeddings of exercise-answering data. The proposed Bi-LSTM model typically comprises an embedding layer, dropout layer, bidirectional LSTM layer, attention layer, and output layer. The Bi-LSTM model includes multiple attention modules.

#### Embedding layer.

Let’s consider a set of phrases denoted as $\left\{s=s_{1},s_{2},...,s_{n}\right\}$, where each sentence has a set of words $w=\{w_{1},w_{2},...,w_{n}\}$. The sentence can be represented as an embedding ($x_{t}=\{x_{1},x_{2},...,x_{n}\}$), which can then be used to create the embedding layers of the Bi-LSTM network. The word embeddings utilized in this investigation are produced via the pre-trained GloVe embeddings. The embeddings possess a standardised dimension of 100.

#### Dropout layer.

The dropout layer [47] in the proposed Bi-LSTM models serves the objective of alleviating the issue of over-fitting [48] in deep learning architectures.

#### Bi-LSTM layer.

A typical Bi-LSTM model consists of two consecutive LSTM layers [46], which function in opposite directions to handle knowledge states in both the forward and backwards ways. The suggested model consists of a total of two hidden layers of Bi-LSTM. The forward LSTM and backwards LSTM components in a Bi-LSTM model can be mathematically represented as:

- Forward LSTM (as shown in Eq (1)):
  $${f_{t}}^{\to }=\sigma (W_{f}.x_{t}+U_{f}.h_{t-1}+b_{f}),$$ (1)
  where the symbol *x*_*t*_ denotes the present input, *h*_*t*−1_ represents the hidden state, *W*_*f*_ and *U*_*f*_ are the matrices used for weighting in the forget gate, and *b*_*f*_ is the bias term used. The formulation is shown in Eq (2).
  $${i_{t}}^{\to }=\sigma (W_{i}.x_{t}+U_{i}.h_{t-1}+b_{i}),$$ (2)
  where the function $\sigma (x)=\frac{1}{1+e^{-x}}$ defines the sigmoid function. The variables *W*_*i*_ and *U*_*i*_ represent the weighting matrices for the input gate, while *b*_*i*_ indicates the corresponding bias.
  In the next layer, the operation can be formulated in Eq (3):
  $${c_{\sim t}}^{\to }=tanh(W_{c\sim }.x_{t}+U_{c\sim }.h_{t-1}+b_{c\sim }),$$ (3)
  where the weighting matrices used in the feature extraction process are denoted as $W_{c\sim }$ and $U_{c\sim }$, and the corresponding bias is indicated as $b_{c\sim }$. The function *tanh*(*x*) can be equivalently represented as $\frac{1-e^{-2x}}{1+e^{-2x}}$, which is worth noting.
  As shown in Eq (4):
  $${o_{t}}^{\to }=\sigma (W_{o}.x_{t}+U_{o}.h_{t-1}+b_{o}),$$ (4)
  where the variables *W*_*o*_ and *U*_*o*_ denote the weighting matrices employed in the output gate, whilst *b*_*o*_ denotes the corresponding bias.
  As shown in Eq (5):
  $${c_{t}}^{\to }=i_{t}⊙c_{\sim t}+f_{t}⊙c_{t-1},$$ (5)
  where ⊙ denotes the operator.
  As shown in Eq (6):
  $${h_{t}}^{\to }=tanh(c_{t})\times o_{t},$$ (6)
  The LSTM model [46] represents the forget gate, input gate, and output gate as equations Eq (1), Eq (2), and Eq (4), respectively. The equation denoted as Eq (3) is employed to demonstrate the process of generating embeddings.
- Backward LSTM:
  $${f_{t}}^{\leftarrow }=\sigma (W_{f}.x_{t}+U_{f}.h_{t+1}+b_{f}),$$ (7)
  $${i_{t}}^{\leftarrow }=\sigma (W_{i}.x_{t}+U_{i}.h_{t+1}+b_{i}),$$ (8)
  $${c_{\sim t}}^{\leftarrow }=tanh(W_{c\sim }.x_{t}+U_{c\sim }.h_{t+1}+b_{c\sim }),$$ (9)
  $${o_{t}}^{\leftarrow }=\sigma (W_{o}.x_{t}+U_{o}.h_{t+1}+b_{o}),$$ (10)
  $${c_{t}}^{\leftarrow }=i_{t}⊙c_{\sim t}+f_{t}⊙c_{t+1},$$ (11)
  $${h_{t}}^{\leftarrow }=tanh(c_{t})\times o_{t},$$ (12)
  The equations labelled as Eq (7), Eq (8), Eq (9), Eq (10), Eq (11), and Eq (12) are utilized to represent the forget gate, input gate, feature extraction module, and output gate in the backwards process.

Furthermore, the combined hidden state of the Bi-LSTM model is acquired by merging the forward and backwards hidden states, as seen in Eq (13).

$${h_{t}}^{↔}={h_{t}}^{\to }⊕{h_{t}}^{\leftarrow }.$$ (13)

#### Output layer.

The softmax activation function was utilized to generate the label for each input textual emotion embedding. Mathematically, it is formulated as Eq (14):

The softmax activation function was employed to produce the label for each input textual emotion embedding. Mathematically, the formulation is expressed as Eq (14):

$$y_{bilstm}=softmax(W_{o}\times o+b_{o}),$$ (14)

where the variable *y*_*bilstm*_ reflects the outcome of the suggested Bi-LSTM models, whereas *o* denotes the output of the last hidden vector of the Bi-LSTM models.

### Informer network

The informer model being suggested has three important characteristics. Initially, it is important to note that a probability attention mechanism offers significantly reduced computational complexity and memory use compared to the traditional transformer model [31]. Furthermore, the distilling process is employed to emphasise dominant components by utilising a cascading layer structure. Furthermore, the generative style decoder has the ability to forecast the entire long sequence output in a single forward operation, rather of doing it step by step. The time stamp of each exercise-answering is included in the input of the suggested informer network.

#### Probability attention mechanism.

**Fig 3:**
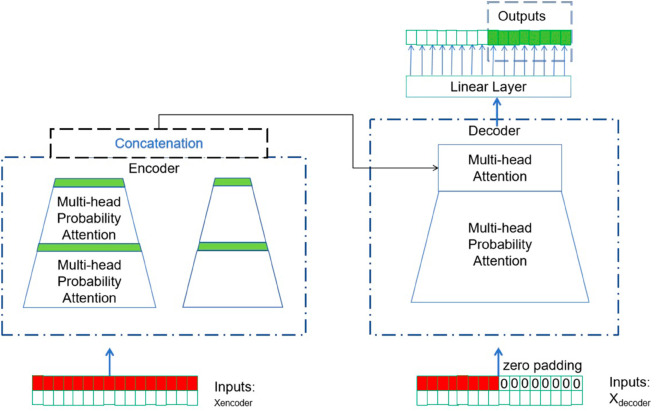
The overall structure of the proposed Informer model.

**Fig 4:**
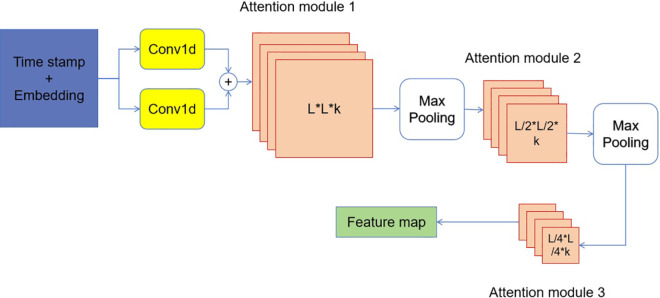
The suggested pipeline utilizes a probability attention method. The variable *L* represents the length of the Conv1d procedure. *k* is the quantity of feature maps produced in every attention module.

The informer, as shown in Fig 3, utilises the probability attention mechanism, as indicated in Fig 4. The use of attention mechanism has been extensively employed in NLP activities in the past few decades. The integration of attention mechanism into neural networks has shown promising outcomes in machine translation and text comprehension. An attention module can be used to emphasise the crucial components in the context that are relevant to the appropriate machine learning activity. In addition, the study conducted by Vaswani et al. [31] made major advancements in the attention mechanism. This study use the multi-head probability attention module, which consists of many heads, to reduce the computational complexity of the canonical attention module described in [31].

The self-attention mechanism described by Vaswani et al. [31]in their work may be expressed mathematically as a tuple input (as shown in Eq (15)) consisting of query, key, and value. This mechanism performs a scaled dot-product operation.

$$𝔸(Q,K,V)=softmax(\frac{QK^{T}}{\sqrt{d}})V,$$ (15)

where $Q\in {\mathbb{R}}^{L_{Q}\times d}$, $K\in {\mathbb{R}}^{L_{K}\times d}$, $V\in {\mathbb{R}}^{L_{V}\times d}$, and *d* denotes the dimension of input.

The original attention mechanism, as demonstrated in the study [31], has proven to be effective in predicting time-series data. Nevertheless, it is plagued by quadratic computational complexity and excessive memory utilization. In order to tackle these issues, a substantial body of research in the field has developed various encoder-decoder architectures. For example, in the probability self-attention module described in [26], each key is limited to attending only to the most influential questions (as seen in Eq (16)).

$$𝔸(Q,K,V)=softmax(\frac{\hat{Q}K^{T}}{\sqrt{d}})V,$$ (16)

where the symbol $\hat{Q}$ represents a sparse matrix that has the same dimensions as matrix *Q*. It contains the most important queries based on the sparsity measurement described in the reference [26]. The utilization of the multi-head architecture allows this probability attention mechanism to produce distinct sparse query-key pairs for each head, hence preventing significant loss of information.

#### Distilling operation.

With the inclusion of the probability attention mechanism, the encoder’s feature map contains redundant combinations of value V. The distillation process is employed to enhance the prominent characteristics in the feature map and produce a concentrated feature map in the subsequent layer, as depicted in Fig 4. This operation has the ability to reduce the time dimension of the inputs, and this may be expressed mathematically as (as demonstrated in equation Eq (17)):

$$X_{j+1}^{t}=MaxPooling(ELU(Conv1D([X_{j}^{t}{]}_{A}))),$$ (17)

where the notation $[X_{j}^{t}{]}_{A}$ represents the attention block, *Conv*1*D*(.) means the one-dimensional convolutional operation with a kernel width of 5 in the temporal dimension, and *ELU*(.) refers to the activation function described in the paper by Clevert et al. [49]. The max-pooling layer, with a stride of 2, can decrease the size of *X*_*t*_ by half, resulting in a significant reduction in memory use. The self-attention distilling layer progressively reduces one layer at a time, like a pyramid as depicted in Fig 4. The final hidden representation of the encoder is obtained by combining the outputs of all the stacks.

#### Generative decoder.

The decoder employed in the suggested methodology is influenced by the research conducted by Vaswani et al. [31], since it comprises of two multi-head attention layers. Meanwhile, the generative method is utilized to achieve lengthy sequence prediction. The input of the decoder is expressed as (as illustrated in Eq (18)):

$$X_{decoder}^{t}=Concat(X_{token}^{t},X_{O}^{t})\in {\mathbb{R}}^{\left(L_{token}+L_{y}\right)}\times d_{model},$$ (18)

where the variable $X_{token}^{t}$ is a matrix of size $L_{token}\times d_{model}$ and represents the initial token. On the other hand, $X_{O}^{t}$ is a matrix of size $L_{y}\times d_{model}$ and serves as a placeholder for the anticipated sequence. The probability attention module can effectively avoid auto-regressive by utilizing the masking attention method. The informer network’s ultimate output is obtained through a linear layer, with the size of the layer, denoted as *d*_*y*_, depending on whether it is used for univariate prediction or multivariate result.

## Results

This section provides an overview of the datasets utilized, the assessment metrics employed, the experimental conditions, and the outcomes of the suggested methodology.

### Dataset

The details of the exploited public datasets in this study are available in Table 1 ((1) Assistments Data Mining Competition 2009 (Assistments2009). The data is accessible at the following link: https://sites.google.com/site/assistmentsdata/home/; (2) Assistments Data Mining Competition 2017 (Assistments2017). The data is accessible at the following link: https://sites.google.com/view/assistmentsdatamining/data-mining-competition-2017; (3) EdNet. The data is accessible at the following link: https://github.com/riiid/ednet). Initially, the data set known as Assistments2009 was collected via the ASSISTments online tutoring platform over the year of 2009 to 2010. Assistments2017 was derived from the ASSISTments data mining competition that took place in 2017. EdNet is a substantial dataset that is available in [50]. The dataset comprises 130 million records derived from 0.78 million pupils. In order to streamline the experimental method, this study exclusively utilizes the data from 4,000 students. In this study, the absolute time is used for each student answering record. These timestamps, in standard date-time format, can be used to identify the order of learning events. Before model input, the timestamps were normalized using Min-Max normalization into the range 0-1. For these three datasets, 80% of the data is used for training, while the remaining 20% is used for testing.

**Table 1:** The details of the publicly available dataset.

Dataset	Total records (No.)	Total exercises (No.)	Students (No.)
Assistments2009	251,000	16,891	4,151
Assistments2017	842,000	3,162	1,709
EdNet	201,000	10,175	4,000

### Dataset pre-processing

Before feeding the data into the proposed model for knowledge tracing, comprehensive dataset pre-processing was essential.

#### Data cleaning.

We meticulously addressed potential issues in the original datasets, including missing values and outliers. For missing values, the proposed approach was data-characteristic-driven. In the case of numerical features like answering time, when the proportion of missing values was low, the mean imputation was used.

#### Data encoding.

Given the abundance of categorical data in our datasets, such as question types and student identities, and the fact that computers operate on numerical data, we implemented multiple encoding techniques. For categorical variables with a finite number of unordered categories, One-Hot Encoding was used to convert each category into a binary vector for accurate computer recognition.

#### Normalization.

Additionally, continuous features such as answering time were normalized, through Min-Max normalization to the range [0, 1] to have a mean of 0 and a standard deviation of 1. This normalization step aimed to enhance the training efficiency and performance of the proposed model.

### Implementation settings

For hardware, we use 4 NVIDIA GeForce RTX 4090 GPUs. Each computer is equipped with 32GB of RAM. As for the CPU, we choose an Intel Xeon Gold 6338 which is quite suitable for handling the associated data processing tasks. In the training process, we employed a series of strategies alongside the enhanced Adam optimizer [51]. This optimizer was utilized with a carefully chosen epsilon value of 1e-9 to maintain numerical stability, automatic mini-batching to efficiently process large datasets, and a dropout rate of 0.1 for each model to introduce regularization and prevent complex co-adaptations on training data. To further address overfitting, we implemented early stopping (set to 10 in our experiments), monitoring the validation loss during training and halting the process if it ceases to decrease, indicating that the model might be memorizing the training data rather than learning generalizable patterns. Additionally, we employed L2 regularization, which adds a penalty for large weights to the loss function, encouraging the model to maintain smaller weights and thus reducing overfitting. Each model was trained with a unique set of epochs and learning rates tailored to their specific architecture and complexity. The word embeddings for the models were produced using GloVe, a pre-trained word embedding model known for its effectiveness in capturing semantic relationships. The embeddings were generated within a 200-dimensional space, trained for 200 iterations, and a learning rate of 0.001 was employed to ensure the convergence of the embeddings. In the experiments, the hyperparameter optimization for the LSTKT model was conducted using grid search, which is a robust method to explore predefined parameter ranges. Key parameters tested in this process included dropout rate, learning rate, and Bi-LSTM hidden layers. In general, the hyper-parameters used in this work are listed in Table 2.

**Table 2:** The hyper-parameter settings used in this study.

Parameter	Setting
optimizer	Adam (ϵ=1e−9)
dropout rate	0.1
Learning rate	0.001 (Bi-LSTM) or 0.002 (Informer)
Epochs	200
Early stop	10

To note that for the Assistments2009 dataset, each epoch took 12.4 minutes to complete. When training on the Assistments2017 dataset, each epoch took around 18.9 minutes. For the EdNet dataset, each epoch had a training time of 21.6 minutes.

**Fig 5:**
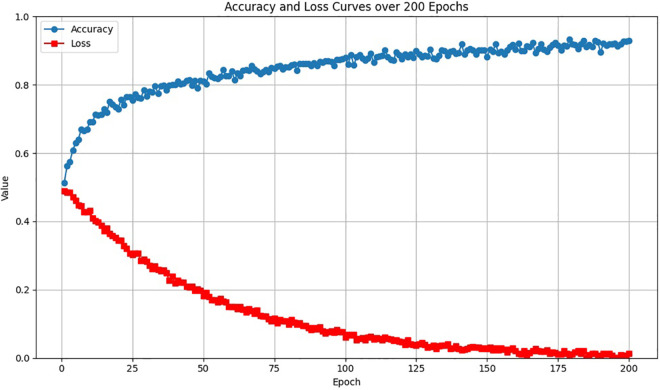
The accuracy and loss curves of the proposed approach in the training process.

In the training process of this study, the presentation and analysis of the model’s performance are of great significance. Fig 5 depicts the accuracy and loss curves over 200 epochs, which effectively mirror the model’s training process.

As shown in Fig 5, at the commencement of training, the model’s accuracy hovers around 50%. As the training unfolds, the accuracy initially surges. The addition of random noise to the data gives rise to minor fluctuations, emulating the real-world intricacies during model training. In the later phase, the growth rate decelerates and eventually plateaus at approximately 92.5%, signifying that the model has gradually assimilated the data characteristics and its performance has achieved stability. Concerning the loss curve, at the start of training, the loss value stands as high as 0.5. Initially, the loss value drops precipitously. Owing to the random noise incorporated, the curve exhibits small fluctuations. As training progresses, the rate of decline gradually slackens until it ultimately stabilizes at 0.01. This indicates that the disparity between the model’s predicted values and the true values is steadily diminishing, and the model’s fitting efficacy is continuously improving. Overall, these two curves comprehensively illustrate the entire training process of the model, from its initial state of instability to gradual convergence and performance enhancement.

### Evaluation metrics

Typically, we use accuracy (as demonstrated in Eq (19)) and AUC [52] as the metrics for evaluation. In general, the mathematical expression of accuracy is provided in Eq (19). To obtain the AUC value, as mentioned by Zhu et al. [52], TPR and FPR are computed for each possible threshold, as shown in Eq (20) and Eq (21). Then, the points (FPR, TPR) are drawn with FPR as the x-axis and TPR as the y-axis in the coordinate system. Finally, the AUC value can be approximated by numerical integration method.

$$Accuracy=\frac{TP+TN}{TP+TN+FP+FN},$$ (19)

where TP, TN, FP, and FN symbolize the following: TP stands for true positive, TN stands for true negative, FP stands for false positive, and FN stands for false negative.

$$TPR=\frac{TP}{TP+FN}$$ (20)

$$FPR=\frac{FP}{FP+TN}$$ (21)

Furthermore, to assess the effectiveness of the suggested method in predicting long sequences, both mean squared error (MSE) and mean absolute error (MAE) are employed.

### Comparison experimental results

#### Comparing with the KT methods.

To evaluate the efficacy of the proposed model for short-term forecasting, we employed state-of-the-art algorithms in the comparison testing. The state-of-the-art approaches include BKT [8], KTM [53], DKT [11], DKVMN [54], SAKT [55], AKT [32], CoKT [56], GKT [39], CoSKT [57], GRKT [74], and FlucKT [75]. The comparative findings of the proposed models on the dataset can be seen in Table 3.

**Table 3:** The comparison between the state-of-the-arts and the proposed models on the datasets.

Method	Assistments2009		Assistments2017		EdNet	
	Acc. (p value)	AUC	Acc. (p value)	AUC	Acc. (p value)	AUC
BKT	0.6974 (0.04)	0.7015	0.6158 (0.02)	0.6239	0.6253 (0.03)	0.6392
KTM	0.6318 (0.02)	0.6269	0.6127 (0.05)	0.6243	0.6124 (0.05)	0.6735
DKT	0.6738 (0.07)	0.7052	0.7083 (0.04)	0.6449	0.6374 (0.03)	0.6361
DKVMN	0.7112 (0.03)	0.7258	0.6430 (0.04)	0.6566	0.6328 (0.01)	0.6422
SAKT	0.7238 (0.02)	0.7419	0.6539 (0.04)	0.6626	0.5963 (0.05)	0.6336
AKT	0.7272 (0.01)	0.7468	0.6901 (0.03)	0.7055	0.6649 (0.04)	0.7056
CoKT	0.7264 (0.02)	0.7303	0.6699 (0.05)	0.7014	0.6156 (0.05)	0.6437
GKT	0.7325 (0.01)	0.7409	0.6854 (0.04)	0.7016	0.6245 (0.05)	0.6876
CoSKT	0.7629 (0.11)	0.7763	0.7236 (0.08)	0.7015	0.6538 (0.04)	0.6561
GRKT	0.7398 (0.02)	0.7914	0.7219 (0.05)	0.7268	0.6714 (0.05)	0.7015
FlucKT	0.7564 (0.03)	0.7857	0.7255 (0.01)	0.7183	0.6649 (0.05)	0.6841
LSTKT	0.7849 (-)	0.7881	0.7422 (-)	0.7282	0.6817 (-)	0.7078

**Fig 6:**
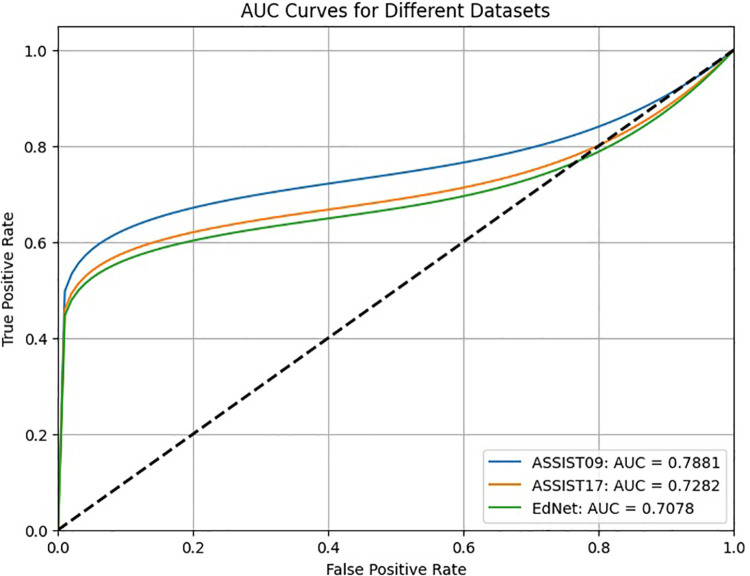
The ROC curves of the proposed approach on the Assistments2009, Assistments2017, EdNete datasets.

The Bi-LSTM and informer model demonstrated superior performance, achieving an accuracy of 78.49% and an AUC of 78.81% for the Assistments2009 dataset, an accuracy of 74.22% and an AUC of 72.82% for the Assistments2017 dataset, and an accuracy of 68.17% and an AUC of 70.78% for the EdNet dataset. The ROC curves are provided in Fig 6.

**Fig 7:**
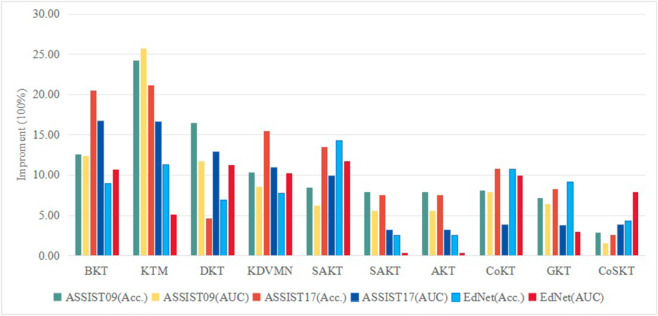
The superiority of the proposed approach compared to the current state-of-the-art algorithms. The proposed strategy has demonstrated higher performance compared to other methods in terms of accuracy and AUC on three distinct datasets.

In addition, the superiority of the proposed strategy compared to the most advanced techniques is demonstrated in Fig 7.

Overall, deep learning techniques described in the literature have consistently outperformed shallow learning models when it comes to short-term prediction. This demonstrates the benefits of using deep learning models to capture the internal structure of students’ knowledge states in knowledge tracing applications. Nevertheless, superficial learning techniques might still hold significant worth in some applications. For example, KTM [53] demonstrates superior performance in terms of accuracy and AUC value on three datasets. Furthermore, the graph-based approach, shown by GKT [39], incorporates the connections between KCs to construct a graph. Furthermore, GKT has the capability to produce similar outcomes as the suggested method. Hence, the previous information based on graphs can be utilized to enhance the suggested model even more.

#### Comparing with the predicting methods.

In order to assess the effectiveness of the model in predicting extended sequences, we employed state-of-the-art algorithms in our comparative tests. The advanced algorithms now used are ARIMA [58], Prophet [59], LSTMa [60], LSTnet [61], and DeepAR [62]. The comparison outcomes of the proposed models on the dataset may be located in Table 4.

**Table 4:** The comparison between the state-of-the-arts and the proposed models on the datasets.

Method	Assistments2009		Assistments2017		EdNet	
	MSE	MAE	MSE	MAE	MSE	MAE
ARIMA	0.807	0.759	1.154	0.457	2.258	1.364
Prophet	0.320	0.421	0.648	0.657	1.976	2.483
LSTMa	0.579	0.687	1.434	0.878	1.506	1.118
LSTnet	0.972	1.412	1.072	0.751	3.170	4.539
DeepAR	0.201	0.376	0.325	0.419	0.667	0.718
LSTKT	0.197	0.284	0.239	0.364	0.559	0.587

As demonstrated in Table 4, the proposed approach has achieved superior peformance over the state-of-the-art techniques. In addition, DeepAR also achieves promising outcomes in terms of accuracy and AUC across the three available datasets. The main factor can be attributed to the matching deep learning architecture.

### Ablation study

**Fig 8:**
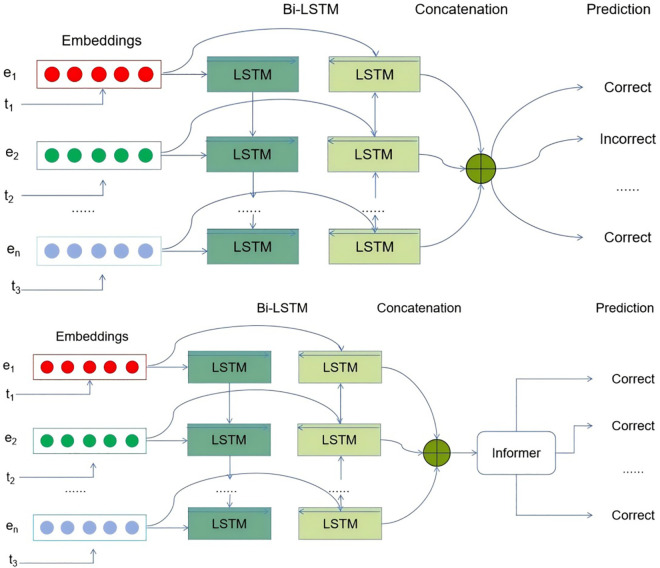
The suggested approach includes two alternate architectures for the Bi-LSTM models: the Bi-LSTM model (Top) and the Bi-LSTM with a single informer network (Bottom).

To delve deeper into the individual contributions and significance of each component within the proposed model, we conducted a comprehensive series of ablation studies. These investigations are pivotal for understanding the distinct roles and the cumulative impact of the various elements that constitute our model. The LSTKT model, which stands at the core of our research, was systematically deconstructed and its structure was alternately substituted with two distinct design configurations. These alternative designs were crafted to isolate the effects of specific architectural choices and to evaluate their individual merits and drawbacks. The modifications were meticulously documented and visually represented in Fig 8, which provides a clear comparative framework.

**Fig 9:**
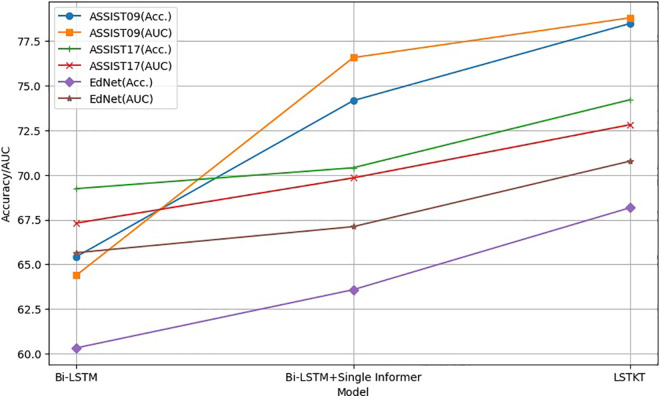
The comparison results between the LSTKT models with two different architectures.

The findings of comparing the LSTKT models with two distinct architectures are presented in Fig 9.

**Fig 10:**
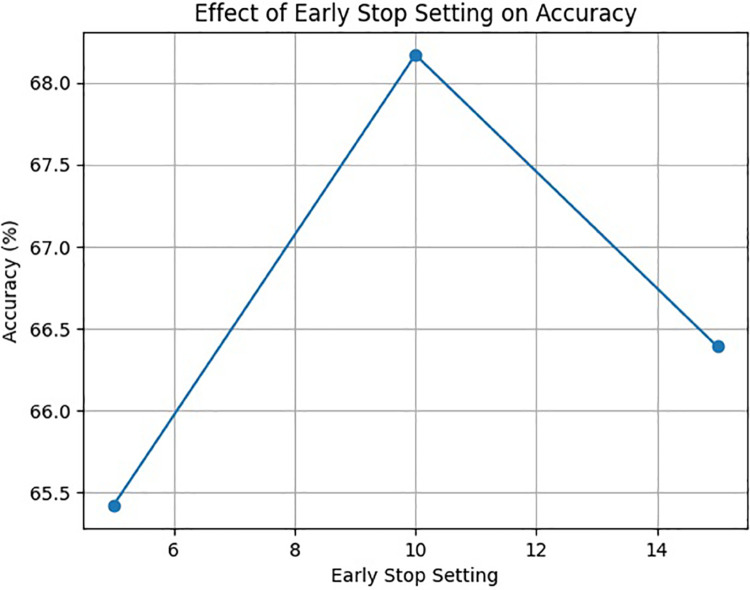
The relationship between different early stop settings and the corresponding accuracy of the LSTKT model on the EdNet dataset.

To further validate the effectiveness of the proposed model’s parameter settings, we conducted the ablation studies on two crucial hyperparameters: the early stopping and the dropout rate. Early stopping is a crucial technique in preventing overfitting during the training process. By varying the patience parameter, its impact on the model’s performance was evaluated. In general, the values of 5, 10, and 15 were tested. This approach not only helped to fine-tune the training process but also provided insights into how the model’s generalization ability was affected by different stopping points. The results of this ablation study demonstrated that an early stop setting with a patience of 10 led to the best performance in terms of the model’s ability to handle long-sequence time-series prediction for knowledge tracing, as shown in Fig 10.

In addition, dropout is a regularization technique that helps prevent overfitting by randomly deactivating neurons during training. We tested dropout rates of 0.0, 0.1, 0.2, 0.3, and 0.4. As presented in Table 5, the optimal dropout rate of 0.1, as used in the main experiments, effectively mitigated overfitting while maintaining the model’s learning capacity, leading to better generalization performance.

**Table 5:** Dropout rate ablation study results on the EdNet dataset.

Dropout Rate	AUC
0.0	0.6742
0.1	0.7078
0.2	0.6872
0.3	0.6868
0.4	0.6823

## Discussion

The proposed deep learning model, which integrates Bi-LSTM and Informer networks, presents a significant advancement in the field of knowledge tracing. The integration of bidirectional LSTM with Informer networks has yielded a model that is not only capable of capturing the intricate temporal dynamics of learning but also adept at generalizing across various educational contexts. The bidirectional flow of information in Bi-LSTM allows for a more nuanced understanding of a student’s learning trajectory, while the Informer network’s optimized complexity ensures that the model remains computationally feasible. This balance between depth and efficiency is crucial for practical applications in educational technology. The proposed approach outperforms existing models in terms of predictive accuracy and AUC. The main findings highlight the model’s ability to process large volumes of educational data without compromising on the granularity of analysis. This is particularly important in educational settings where long-term and accurate feedback can significantly influence learning outcomes.

Despite the promising results, the study still has the following limitations. Firstly, the model’s performance is highly reliant on the quality and representativeness of the input data. Poor-quality data or data that fails to adequately represent real-world scenarios may significantly degrade the model’s performance, thus affecting the reliability and practicality of the research results. Secondly, although the complexity of the Informer network has been reduced to some extent, there remains room for further optimization. Especially when dealing with the vast and diverse datasets in real-world educational environments, the current network architecture may not be able to handle them efficiently, and further improvements are required to meet the needs of large-scale data processing.

## Conclusion

In this study, a novel deep learning approach has been proposed that synergistically combines Bi-LSTM and Informer networks for knowledge tracing. The proposed method leverages a Bi-LSTM network to meticulously extract sequential information from students’ records, capturing both forward and backward dependencies within the learning process. Subsequently, the refined pipeline feeds the output of the Bi-LSTM into an Informer network, which has been optimized to reduce computational complexity significantly while maintaining its efficacy in handling long-range dependencies.

The integration of Bi-LSTM and Informer networks has demonstrated a substantial improvement in the accuracy of knowledge tracing models. The bidirectional nature of Bi-LSTM provides a comprehensive understanding of the learning sequence, while the Informer network, with its reduced complexity, efficiently processes this information to predict knowledge levels and learning outcomes. One of the key advancements in this approach is the reduction of the Informer network’s complexity. By optimizing the probability self-attention mechanism, the model achieves a balance between performance and computational efficiency, making it more suitable for large-scale applications.

The proposed model opens avenues for further research and development. Future work could focus on enhancing the model’s interpretability to provide educators with actionable insights into students’ learning trajectories. In addition, exploring the integration of multimodal data, such as combining textual, behavioral, and physiological data, could offer a more holistic view of the learning process. Furthermore, investigating adaptive learning strategies based on the model’s predictions could lead to personalized learning experiences that are dynamically tailored to each student’s needs.
